# EGF receptor inhibitors comprehensively suppress hepatitis B virus by downregulation of STAT3 phosphorylation

**DOI:** 10.1016/j.bbrep.2020.100763

**Published:** 2020-04-18

**Authors:** Chong J. Gan, Wen F. Li, Chun N. Li, Ling L. Li, Wen Y. Zhou, Xiao M. Peng

**Affiliations:** a Center of Infectious Diseases, The Fifth Affiliated Hospital, Sun Yat-Sen University, Zhuhai, Guangdong, 519000, China; b Central Laboratory, The Fifth Affiliated Hospital, Sun Yat-Sen University, Zhuhai, Guangdong, 519000, China

**Keywords:** Hepatitis B virus, Antiviral therapy, Epidermal growth factor receptor inhibitor, STAT3, Covalently closed circular DNA, HBV, hepatitis B virus, HCC, hepatocellular carcinoma, NAs, nucleotide/nucleoside analogues, IFN, interferon, cccDNA, covalently closed circular DNA, HNF3, hepatocyte nuclear factor 3, STAT3, signal transduction and activators of transcription 3, EGF, epidermal growth factor, EGFR, epidermal growth factor inhibitor, NTCP, sodium taurocholate cotransporting polypeptide, GEq, genome equivalent, PCR, polymerase chain reaction, SOCS3, suppressor of cytokine signaling 3, MTT, 3-(4,5-dimethyl-2-thiazolyl)-2,5-diphenyl-2-H-tetrazolium bromide, HBsAg, hepatitis B surface antigen, HBeAg, hepatitis B e antigen

## Abstract

Current antiviral therapy can not cure chronic hepatitis B virus (HBV) infection or eliminate the risk of hepatocellular carcinoma. The licensed epidermal growth factor receptor (EGFR) inhibitors have found to inhibit hepatitis C virus replication via downregulation of signal transducers and activators of transcription 3 (STAT3) phosphorylation. Since STAT3 is also involved in HBV replication, we further studied the anti-HBV efficacy of the EGFR inhibitors in this study. HBV-transfected HepG2.2.15 cells and HBV-infected HepG2-NTCP cells were used as cell models, and HBV replication, the syntheses of viral antigens and the magnitude of the covalently closed circular DNA (cccDNA) reservoir were used as indictors to test the anti-HBV effects of EGFR inhibitors erlotinib and gefitinib. Erlotinib inhibited HBV replication with a half-maximal inhibitory concentration of 1.05 μM. It also reduced the syntheses of viral antigens at concentrations of 2.5 μM or higher. The underlying mechanism was possibly correlated with its inhibition on STAT3 phosphorylation via up-regulation of suppressor of cytokine signaling 3. Gefitinib also inhibited HBV replication and antigen syntheses. Compared with the commonest antiviral drug entecavir, these EGFR inhibitors additionally reduced hepatitis B e antigen and erlotinib also marginally affected the cccDNA reservoir in HBV-infected HepG2-NTCP cells. Interestingly, these promising anti-HBV effects were significantly enhanced by extension of treatment duration. In conclusion, EGFR inhibitors demonstrated a comprehensive anti-HBV potential, highlighting a new strategy to cure HBV infection and suggesting animal model-related studies or clinical try in the future.

## Highlights

• We test the anti-HBV potential of EGFR inhibitors erlotinib and gefitinib *in vitro.*
• Erlotinib inhibits HBV replication in clinically safe concentrations.
• EGFR inhibitors suppress HBV by downregulation of STAT3 phosphorylation.
• EGFR inhibitors show a comprehensive anti-HBV potential including the cccDNA.

## 1 Introduction

Hepatitis B virus (HBV) infection is a leading cause of hepatocellular carcinoma (HCC) and liver cirrhosis [1]. Antiviral therapy employing either nucleotide/nucleoside analogues (NAs) or recombinant interferon (rIFN) -α has been significantly improved the prognosis of HBV infection [2]. However, it is urgent to search for new anti-HBV strategies since the cure of the infection is seldom achieved and the persistent suppression of viral replication below the limit of detection does not eliminate the risk of HCC development [3,4].

HBV uniquely establishes a reservoir of covalently closed circular DNA (cccDNA) in the nuclei of infected hepatocytes. The residual HCC risk of current antiviral therapy is thought to be contributed by the persistent viral replication and antigen production due to the long-term existence of the cccDNA reservoir [5]. The cccDNA is organized into a minichromosome to serve as the template for the transcriptions of all viral messenger RNAs including a genome-sized pregenomic RNA that is reversely transcribed into open circular duplex DNA at last. The transcription of pregenomic RNA is controlled by the basal core promoter that is profoundly influenced by two enhancers, EN I and EN II. EN I consists of multiple transcription factor binding sites. Among these sites, two adjacent sites of hepatocyte nuclear factor 3 (HNF3) and signal transducers and activators of transcription 3 (STAT3) are noticeable. They combine with the complex of HNF3 and STAT3 to activate the EN I function [6], which serves as the underlying mechanism of type I interferon to promote HBV replication in mice with a low HBV load [7]. Concordantly, STAT3 inhibition by decoy ODN or siRNA leads to the decreases in HBV replication and viral antigen syntheses though the influence on cccDNA is regrettably not investigated [8,9].

Epidermal growth factor (EGF)-EGF receptor (EGFR) signaling pathway plays key roles in both HCC and liver cirrhosis. EGF expression is up-regulated in cirrhotic liver diseases [10]. A functional polymorphism in the human EGF gene is associated with the increased cirrhotic progression and the elevated risk of HCC development [11]. Moreover, the EGFR gene is correlated with STAT3 expression [12]. A licensed EGFR inhibitor, erlotinib, enhances the ant-HCV activity of rIFN-α by down-regulation of STAT3 phosphorylation [13]. In addition, erlotinib has been found to inhibit the activation of myofibroblastic hepatic satellite cells, prevent the progression of cirrhosis, regress fibrosis and block subsequent development of HCC in rodent models [14]. Since STAT3 is favorable for HBV replication [6], erlotinib or EGFR inhibition may be of anti-HBV efficacy. Together with their HCC and cirrhosis preventing effects [14], EGFR inhibition may serve as a potential option to improve current antiviral therapy of chronic HBV infection.

In this study, we aimed to investigate whether EGFR inhibitors (i) inhibit viral replication and antigen syntheses of HBV and (ii) offer an opportunity to interfere with the radical cure obstacle-related cccDNA reservoir.

## 2 Materials and methods

### 2.1 Cell lines and cell cultivation

HepG2 and HepG2.2.15 cells are the reserves of our laboratory. HepG2-NTCP cells were established, as reported [15], by constructing sodium taurocholate cotransporting polypeptide (NTCP)-lentiviruses-expressing system based on lentiviral expression vector pCDH-CMV-EF1-copGFP-T2A-Puro, infecting HepG2 cells and performing selection using puromycin (Sigma-Aldrich Corporation, St. Louis, MO, USA). All cells were grown in Dulbecco's modified Eagle's medium with 10% (V/V) fetal bovine serum. For HepG2.2.15 and HepG2-NTCP cells, 380 μg/mL geneticin and 2 μg/mL puromycin cells were added to the culture media, respectively.

### 2.2 HBV infection

HBV inoculum was prepared from a patient with serum HBV DNA of 3.5✕10^7^ copies/mL (genotype C). The serum was cleared through a 0.45 μm filter and precipitated with 10% (M/V) PEG8000 and 2.3% (M/V) NaCl. The precipitates were resuspended with medium at about 100 vol of the serum. The final concentration of HBV was quantified by real-time fluorescent polymerase chain reaction (PCR, Taan Gene Company, Guangzhou, China). HepG2-NTCP cells were infected with HBV at 200 genome equivalent (GEq)/cell in the presence of 4% (M/V) PEG8000 for 16 h.

### 2.3 Inhibitor treatments

To study the antiviral effect or cell viability of EGFR inhibitors, HepG2.2.15 cells near confluent growth (>90%) were treated with 0–12.5 μM erlotinib (ab141930, Abcam, Cambridge, UK) or 10 and 20 μM gefitinib (ab142052, Abcam) for 48 h. The media were replaced every 24 h. To study the influence of EGFR inhibitors on STAT3 phosphorylation, HepG2.2.15 cells were treated with or without 10 μM erlotinib for 1 h. To study the anti-HBV effects of EGFR in HBV-infected model, HepG2-NCTP cells infected with HBV (2✕10^2^ GEq/cells) for 6 days before treating with or without 2.5 μM erlotinib, 10 μM gefitinib or 30 nM ETV (Sigma-Aldrich Corporation) for 48 h to insure the effect evaluation at the summit of HBV replication. To study the sustained influences of EGFR inhibitors on viral antigens and the cccDNA reservoir, HepG2-NCTP cells were treated with or without 2.5 μM erlotinib for up to 8 days. Erlotinib, gefitinib and ETV were all dissolved in dimethylsulfoxide (DMSO). The solvent was standardized at the concentration of 0.125% (V/V).

### 2.4 Quantitative PCR

The viral particles (virions) in media were precipitated using polyethylene glycol 8000. The contaminated cellular integrated or plasmid HBV DNA was removed by digestion with DNase I. The core-associated HBV DNA was extracted using phenol-chloroform protocol. The supernatant core-associated HBV DNA was quantitatively examined using real-time fluorescent PCR. The results were normalized to the number of cells. To exam HBV cccDNA, cellular DNA was extracted and digested by plasmid-safe ATP-dependent DNase (Epicentre, Madison, WI, USA) according to the manufacturer's instructions [16]. Real-time fluorescent PCR was performed using HBV cccDNA-selective primers and probe were as follows: forward 5′-ATC TGC CGG ACC GTG TGC-3′, reverse 5′-TTG GAG GCT TGA ACA GTA GGA-3′ and probe 5′-FAM-GCA CGT CGC ATG GAG A-MGB-3’. Sampling was normalized to the cellular DNA.

### 2.5 Southern blot analysis

Intracellular core-associated HBV DNA was detected using Southern blot analysis as reported [17]. Southern blot analysis was performed after 5 μg cellular DNA was separated and transferred onto nylon membranes (Roche Applied Science, Indianapolis, IN, USA). The transferred DNA bands were visualized using digoxigenin-labeled DNA probes, horseradish peroxidase-labeled anti-digoxigenin antibody (Roche Applied Science) and enhanced chemiluminescence reagent (Invitrogen Corporation).

### 2.6 Western blot analysis

Intracellular HBV core and precore proteins and cellular p-STAT3 and suppressor of cytokine signaling (SOCS) 3 were detected using Western blot analysis. Total cellular proteins were prepared by lysing the cells on ice for 30 min in lysis buffer (50 mM Tris, pH 7.4, 150 mM NaCl, 1% [V/V] NP-40, 1% [M/V] SDS, 1 mM phenylmethylsulfonyl fluoride, 10 mg/L aprotinin, 10 mg/L leupeptin). Nuclear and cytoplasmic fractionation was performed according to the manufacture's protocol (ThermoFisher Scientific, Shanghai, China). Equivalent amounts of cellular proteins (20 μg) were separated and transferred onto polyvinylidene fluoride membranes (Millipore Corporation, Billerica, MA, USA). Immunoblot analysis was performed using monoclonal antibodies to p-STAT3 (sc-7993, Tyr 705) and SOCS3 (sc-51699) (Santa Cruz Biotechnology, Santa Cruz, CA, USA) or polyclonal antibodies to HBV core protein (DAKO, Carpinteria, CA, USA) and enhanced chemiluminescence reagent (Invitrogen Corporation). The optical density of interesting bands was calculated using image pro plus 6.0 and showed as the ratio against that of the control of β-actin.

### 2.7 Cell viability and growth assays

Cell viability was evaluated using 3-(4,5-dimethyl-2-thiazolyl)-2,5-diphenyl-2-H-tetrazolium bromide (MTT) assay. The cells after treated with EGFR inhibitors were harvested and counted as usual, and then were seeded on 96-well plates at a density of 1.0 × 10^4^ cells/well. Each well was added into 20 μl sterilized MTT solution (5 mg/mL) in dimethylsulfoxide. The cells were incubated for 4 h before the MTT-containing medium were removed. After the crystallization was dissolved by 150 μl dimethylsulfoxide, the absorbance at 562 nm was measured using microplate reader (ThermoFisher Scientific). Cell growth assay was performed just like cell viability assay except that cell count was conducted before the treatments of EGFR inhibitors.

### 2.8 Elisa

Hepatitis B surface antigen (HBsAg) and hepatitis B e antigen (HBeAg) in media was quantified using commercial kits of chemiluminescence immunoassay (USCNK Life Science Incorporation, Wuhan, China).

### 2.9 Statistical analyses

The differences in supernatant virions, HBsAg, HBeAg and cell viability were analyzed using the Student's *t*-test. The standard deviation (SD) was calculated from three independent experiments that were performed in triplicate. A *P* < 0.05 was considered statistically significant. All statistical analyses were conducted using SPSS software (version 11; SPSS Incorporation, Chicago, IL, USA).

## 3 Results

### 3.1 Erlotinib inhibits HBV replication and viral antigen syntheses in HepG2.2.15 cells

**Fig. 1:**
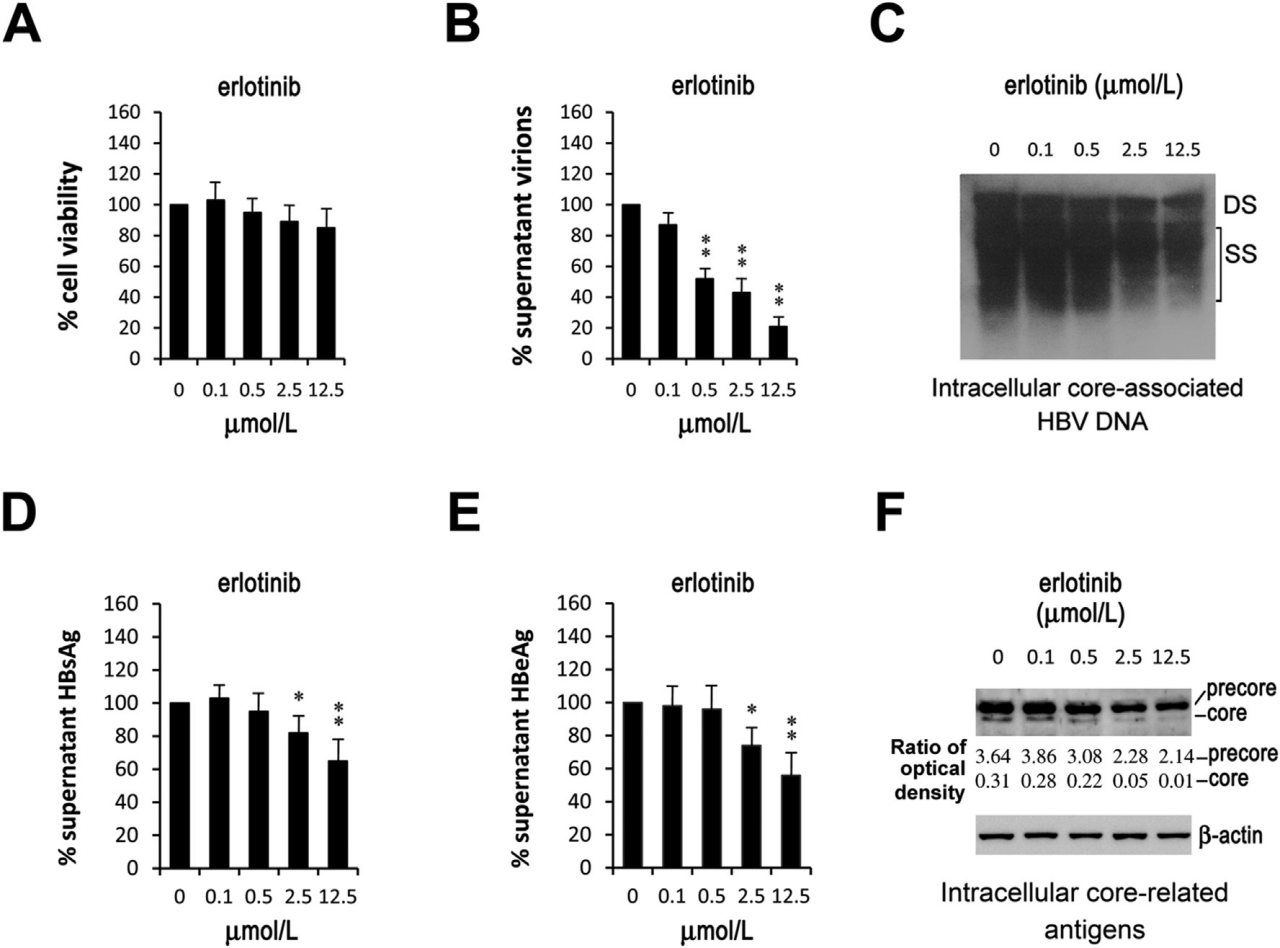
**Erlotinib inhibits HBV replication and viral antigen syntheses.** HepG2.2.15 cells were cultivated with an equal concentration (0.125%) of DMSO and different concentrations (0–12.5 μM) of erlotinib for 48 h. Error bars indicate the SD for three independent experiments that were performed in triplicate. *P < 0.05, **P < 0.01. The intracellular core-associated HBV DNA includes the genomic double-stranded (DS) DNA and the replication intermediate single stranded (SS) DNA. Intracellular core-related antigens include core protein (core) and precore protein (precore). The optical density of interesting bands was showed as the ratio against that of the control of β-actin. (A) HepG2.2.15 cells kept viability of 85% when exposed to erlotinib up to 12.5 μM. Erlotinib inhibited (B) supernatant virions with IC_50_ of 1.05 μM and (C) the intracellular core-associated HBV DNA at concentration of 2.5 μM or higher. The supernatant (D) HBsAg, (E) HBeAg and (F) intracellular core protein were reduced by erlotinib at higher concentrations (2.5 and 12.5 μM).

Erlotinib has been licensed to treat advanced HCC and some type of lung cancer for years. Recently, erlotinib has been found to act synergistically with IFN-α by reducing STAT3 phosphorylation in Huh7.5.1 cell line, a cell model of HCV infection [13]. Due to the importance of STAT3 to HBV replication [6], the influence of erlotinib on HBV was investigated here. HepG2.2.15 is a commonly used HBV-producing cell line. These cells kept a viability of above 85% when exposed to erlotinib at a concentration up to 12.5 μM for 48 h (Fig. 1A). Erlotinib reduced supernatant HBV virions with a half-maximal inhibitory concentration (IC_50_) of 1.05 μM (Fig. 1B). The virion inhibition was accompanied with decreases in intracellular core-associated HBV DNA (Fig. 1C), implying that erlotinib inhibits HBV replication rather than interferes with virion release. Next, we wonder whether erlotinib cover the fatal shortage of current anti-HBV drugs (NAs) to inhibit viral antigen syntheses. Within 48 h, erlotinib at higher concentrations (2.5 and 12.5 μM) significantly decreased supernatant HBsAg and HBeAg (Fig. 1D and E), which accompanied with reduction of intracellular core antigen and precore protein (HBeAg precursor) (Fig. 1F). These results showed that erlotinib inhibited the antigen syntheses of HBV.

### 3.2 Erlotinib inhibits STAT3 phosphorylation and up-regulates SOCS3 expression in HepG2.2.15 cells

**Fig. 2:**
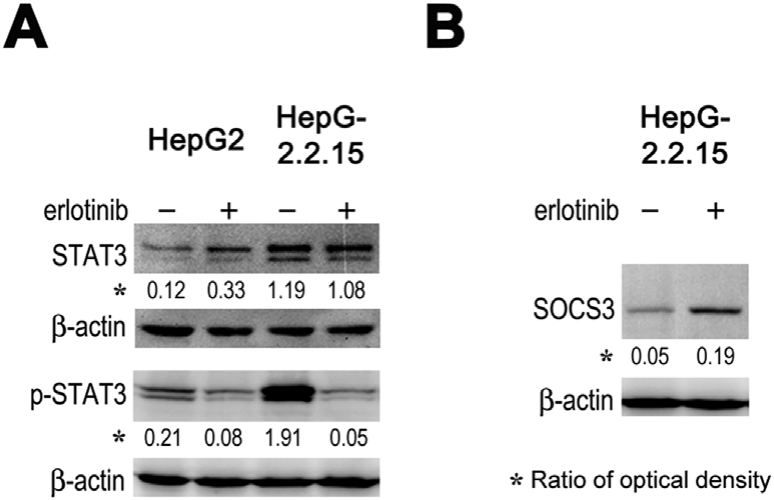
**Erlotinib inhibits STAT3 phosphorylation of and upregulates SOCS3 expression.** HepG2.2.15 cells were cultivated with an equal concentration (0.125%) of DMSO and with or without 10 μM erlotinib for 1 h. STAT3, p-STAT3 and SOCS3 were detected using Western blot analysis. The optical density of interesting bands was showed as the ratio against that of the control of β-actin. (A) HepG2.2.15 cells had much higher levels of STAT3 and p-STAT3 and erlotinib inhibited STAT3 phosphorylation without affection on STAT3 level. (B) Erlotinib increased SOCS3 expression.

Erlotinib demonstrates anti-HCV effect partially through inhibition of STAT3 phosphorylation by induction of the expression of SOCS3, a professional phosphorylation inhibitor of STATs [13,18]. Here, we wondered whether STAT3 was involved in the inhibitory effect of erlotinib on HBV replication. Compared to HepG2 cells, HepG2.2.15 cells had much higher level of STAT3 and p-STAT3. Erlotinib significantly inhibited the phosphorylation of STAT3 without affection on the level of STAT3 (Fig. 2A) and increased the expression of SOCS3 (Fig. 2B). Together with the importance of STAT3 to HBV replication [6,7], these results suggest that erlotinib inhibits HBV replication by inhibition of STAT3 phosphorylation and the upregulation of SOCS3 may be the underlying mechanism to downregulate STAT3 phosphorylation.

### 3.3 EGFR inhibitors demonstrate anti-HBV effects in HBV-infected HepG2-NTCP cells

**Fig. 3:**
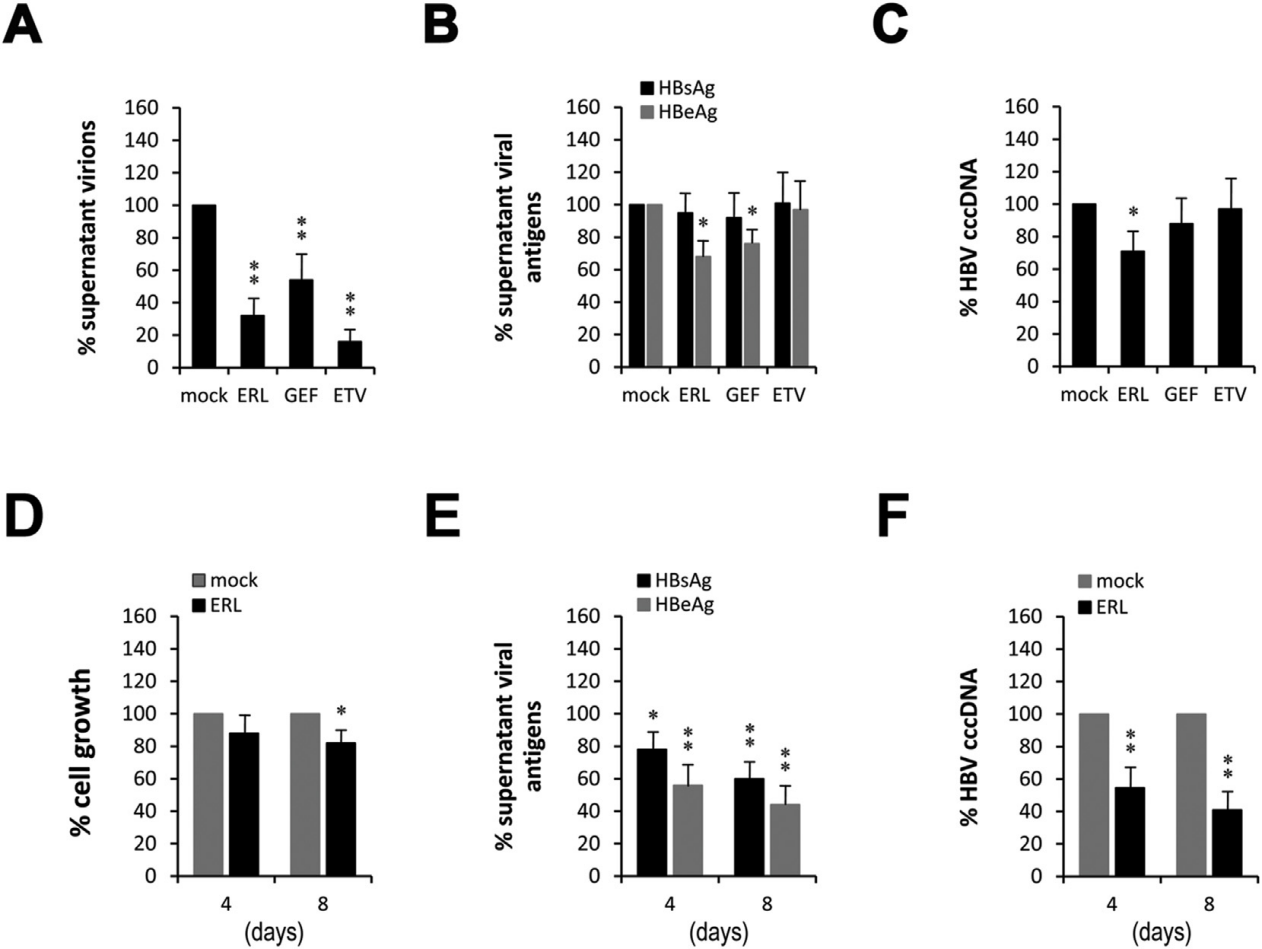
**EGFR inhibitors show anti-HBV efficacy in HBV-infected-model.** HepG2-NTCP cells were infected with HBV (2✕10^2^ GEq/cells) for 6 days before treating with erlotinib (ERL, 2.5 μM), gefitinib (GEF, 10 μM) or entecavir (ETV, 30 nM) for additional 48 h. The solvent DMSO was equally normalized. Error bars indicate the SD for three independent experiments that were performed in triplicate.*P < 0.05, **P < 0.01. (A) Erlotinib, gefitinib and entecavir all significantly inhibited the supernatant virions. (B) Erlotinib and gefitinib, but entecavir, inhibited supernatant HBeAg. (C) Only erlotinib marginally reduced the level of intracellular HBV cccDNA. Prolonged treatment of erlotinib (ERL, 2.5 μM) for 4 and 8 days, respectively, (D) slightly inhibited cell growth (12.4% and 18.2%) and significantly enhanced the inhibitory effects on (E) viral antigens (HBsAg: 22.1% and 40.4%; HBeAg: 43.8% and 56.2%) and (F) intracellular cccDNA (45.4% and 59.3%).

Erlotinib demonstrated anti-HBV effects in the transfected HBV cell model, HepG2.2.15. Next, we evaluate whether erlotinib and another EGFR inhibitor (gefitinib) inhibit HBV replication in HBV-infected cell model, HBV-infected HepG2-NTCP. In order to understand the clinical prospect of EGFR inhibitors, entecavir (ETV), the commonest anti-HBV drug nowadays, is used as positive control. Both EGFR inhibitors (2.5 μM erlotinib and 10 μM gefitinib for 48 h) inhibited the supernatant virions (Fig. 3A). Compared with ETV (30 nM), EGFR inhibitors inhibited viral replication much weaker, but reduced the secretions of HBeAg (Fig. 3B). HBV-infected HepG2-NTCP cells can produce a moderate level of HBV cccDNA [15,19]. It is well known that the persistent existence of the cccDNA is the key factor to affect the cure of HBV infection and ETV with lower cure rate is due to the lack of direct effect on the cccDNA [20]. Excitingly, erlotinib marginally reduced the intracellular level of the cccDNA though the effect of gefitinib was not statistically significant (Fig. 3C). To some extent, both erlotinib and gefitinib demonstrated anti-HBV effects in HBV-infected HepG2-NTCP cells. Erlotinib seemed more effective than gefitinib, which is in concordance with the stronger efficacy of erlotinib on EGFR inhibition.

### 3.4 Prolonged treatment improves the anti-HBV efficacy of erlotinib

Although significantly reduced the supernatant virions, EGFR inhibitors at conventional concentrations only marginally inhibited viral antigens and the cccDNA (Fig. 3A–C). The possible explanation is that the treatment of 48 h may not be longer enough to accumulate the changes in viral antigens and the cccDNA. For this reason, re-tests of the stronger inhibitor erlotinib with prolonged treatments for 4 and 8 days were performed. Due to the anti-proliferative effect of EGFR inhibitors [21], their influence on cell growth was firstly studied. Prolonged treatments with 2.5 μM erlotinib for 4 and 8 days inhibited cell growth at rates of 12.4% and 18.2% (Fig. 3D, respectively, but reduced HBsAg at rates of 22.1% and 40.4% (Fig. 3E), and HBeAg at rates of 43.8% and 56.2% (Fig. 3E), and the cccDNA at rates of 45.4% and 59.3% (Fig. 3F), respectively. These results suggest that the prolonged treatment can be independent of cell growth inhibition to improve the anti-HBV efficacy of erlotinib.

## 4 Discussion

In this study, we found that EGFR inhibitors significantly inhibited HBV replication and moderately reduced viral antigen syntheses in HBV-transfected HepG2.2.15 cells. The underlying mechanism was associated with the decline of STAT3 phosphorylation, possibly via up-regulation of SOCS3 expression. Compared to the commonest antiviral drug ETV, EGFR inhibitors only modestly inhibited HBV replication, but marginally decreased the synthesis of HBeAg and the magnitude of the cccDNA reservoir in HBV-infected HepG2-NTCP cells. Furthermore, prolonged treatment let erlotinib obtain a comprehensive anti-HBV effect including inhibitions to viral replication, antigen syntheses and cccDNA reservoir maintenance. Though further studies are needed, our above results together with the fact that EGFR inhibitors are licensed drugs highlight a promising strategy to overcome the current antiviral challenge of chronic HBV infection.

EGFR is a member (ErbB1) of the ErbB family that contains four receptor tyrosine kinases. It is activated by several ligands including EGF and transforming growth factor-α. Upon ligand binding, EGFR can form homo- or heterodimers with other EGFR family members to activate complex downstream signaling cascades, Ras-Raf-MEK-ERK1/2 and the STAT 3 and 5 pathways [22]. EGF-EGFR signaling pathway has been found to be involved in liver cirrhosis and HCC [10,14]. Its significance to HBV replication, however, is not completely illustrated yet. Here, for the first time, we demonstrated that EGFR inhibitors inhibited the replication, antigen syntheses and cccDNA reservoir of HBV. The underlying mechanism was correlated with inhibition of STAT3 phosphorylation via up-regulation of SOCS3 expression possibly. This mechanism is in concordance with the facts that EGFR overexpression down-regulates SOCS3 expression in mouse embryonic fibroblasts and EGFR inhibitor erlotinib in hepatocytes unleashing SOCS3 expression that inhibits IFN-α-induced STAT3 activity [13,23]. The cccDNA reduction by erlotinib was accompanied with HBV replication and viral antigen synthesis inhibitions, suggesting that erlotinib drains the cccDNA reservoir by limitation of the source.

EGFR inhibitors enhance SOCS3 expression by an intrinsic and ligand-independent effect of EGFR blockage [13]. The viewpoint is further supported by our results that EGFR inhibitors inhibited the cccDNA and viral transcription, which is different from EGF that slightly increases the cccDNA and decreases viral transcription via induction of cell proliferation in duck HBV model [24]. Of course, it will be inevitable that EGFR inhibitors block the signaling of exogenous or endogenous cytokines and growth factor *in vivo*. Therefore, the reduction of cccDNA would be more obvious, whereas the inhibition of viral replication would be weaker based on the results of EGF in duck model [24]. Fortunately, the reverse effect of viral replication is easily eliminated by combination with ETV. Additionally, SOCS3 may counteract the JAK-STAT signaling pathway to affect the host antiviral immune responses [25]. However, preactivation of the JAK-STAT signaling pathway is correlated with nonresponse to rIFN-α therapy [26], and even the blockade of this signaling pathway controls chronic infection of lymphocytic choriomeningitis virus [27]. Based on these facts, there is no need to worry about the possible side-effects of increased SOCS3 in clinical usages. Together with its effects on STAT3, EGFR inhibitors represent a novel class of anti-HBV agents, which are different from interferon and nucleotide/nucleoside analogues.

STATs are a family of seven members that are activated by the signals from cytokine and growth factor receptors in the plasma membrane. They regulate gene transcriptions and play crucial roles in cell proliferation, differentiation, apoptosis, inflammatory response, immunity and angiogenesis. Type I interferon is first discovered to employ STAT1 and STAT2 to inhibit HBV replication. Usually, STAT3 is unique as it is known to direct seemingly contradictory responses [28]. Indeed, HBV activates STAT3 and STAT3 promotes HBV replication [6,7]. The down-regulation of STAT3 results in HBV replication inhibition and reduction of viral antigen syntheses [8,9]. STAT3 promoting HBV replication may involve the well-known HBV replication-enhancing mechanism, STAT3/HNF3γ-EN I enhancer. This mechanism has been reported to play important roles in type I interferon-enhanced HBV replication in mice with a low load of HBV [7]. In this study, EGFR inhibitors were found to suppress HBV replication and viral antigen syntheses to accompany with inhibition of STAT3 phosphorylation, implying that EGFR inhibitors realize anti-HBV via the STAT3/HNF3γ-EN I enhancing mechanism. Moreover, we for the first time demonstrated that STAT3 inhibition significantly reduced intracellular cccDNA. These findings provide additional evidences to show that STAT3/HNF3γ-EN I enhancer is a new anti-HBV target.

STAT3 is commonly constitutively activated in liver and play a key role in tumor formation [23]. The inhibition of STAT3 leads to the suppression of HCC cell lines [8]. EGFR is frequently overexpressed in human HCCs correlating with aggressive tumors, metastasis, and poor patient survival [29]. In addition, EGFR inhibitors demonstrate anti-HCC effect in cell models [30]. These data suggest that EGFR inhibitors are potential as anti-HCC drugs or drugs to prevent HCC. Unfortunately, clinical studies with EGFR inhibitors have so far shown only modest efficacy or no additional effect when used with the currently main drug sorafenib [31]. One possible explanation for treatment failure could be that activating mutations have not been reported in human HCCs [22], perhaps no enough time leaved or intensity of EGFR expression lets EGFR inhibitors take effect. Nonetheless, their values as HCC-prophylactic drugs are still worthy of more studies.

Compared with current antiviral drugs, EGFR inhibitors can directly inhibit HBV replication and viral antigen expressions. Especially, the suppressions of HBsAg and cccDNA highlight the possibility of cure HBV infection. Though the decreases in viral antigens partially results from the cell growth restraint, EGFR inhibitors are still clinically promising since the grow restraint of infected hepatocytes is favorable for the cure of HBV infection as well as the elimination of HCC risk. However, STAT3 is involved in hepatocyte proliferation in partial hepatectomy. EGFR inhibitors may affect the repair of the liver. In addition, EGFR inhibitors metabolisms in liver and excretes from biliary tract, suggesting that there is safety concerns in patients with advanced liver diseases. Indeed, erlotinib has shown hepatotoxicity [32]. Fortunately, the IC50 (1.05 μM) of erlotinib to inhibit HBV replication is less than the plasma concentration (3–4 μM) of the treated patients (150 mg, daily) and recently approved afatinib is much safer. In recent, some reports announce that erlotinib or other EGFR inhibitors cause HBV reactivation in patients with advanced non-small-cell lung cancer in the context with positive HBsAg [33,34]. We prefer to believe it is a hepatotoxic phenomenon since its incidence is low, the onset is much earlier, the ALT/AST ratio is usually about 1 and the damaged hepatocytes may release the trapped HBsAg and HBV DNA to form a pseudo rise in sera. Nonetheless, the efficacy and safety are urged for more studies in animal models before initiating clinical try in the future.

## Author statement

The manuscript was written and formatted in accordance with the latest Instructions for Authors. We certify the following: 1) all of the listed authors have participated actively in the study and have met the requirements for authorship; 2) all of the authors have read and approved the submitted manuscript; 3) the manuscript reports unpublished work which is not currently under consideration elsewhere and will not be submitted to another journal until a final, unfavorable decision has been made by the editors of **BBRC**; and 4) none of the authors has any conflicts of interest with respect to this research.

## Funding

This work was supported by Sun Yat-Sen University (grant number “Five & Five” project), the National Nature Scientific Foundation (grant number 81071366) and the Scientific and Technological Bureau of Guangzhou, Guangdong Province (grant number 201508020059), China.

## Declaration of competing interest

The authors have no conflict of interest to declare.
